# Luminore CopperTouch Surface Coating Effectively Inactivates SARS-CoV-2, Ebola Virus, and Marburg Virus *In Vitro*

**DOI:** 10.1128/AAC.01390-20

**Published:** 2021-06-17

**Authors:** Emily K. Mantlo, Slobodan Paessler, Alexey Seregin, Alfred Mitchell

**Affiliations:** a University of Texas Medical Branch, Galveston, Texas, USA; b Luminore CopperTouch, Houston, Texas, USA

**Keywords:** SARS-CoV-2, COVID-19, Ebola virus, Marburg virus, filoviruses, EPA registration, copper surface, infection control, antiviral, antimicrobial, Luminore CopperTouch antimicrobial touch surfaces

## Abstract

We investigated the ability of Luminore CopperTouch copper and copper-nickel surfaces to inactivate filoviruses and severe acute respiratory syndrome coronavirus 2 (SARS-CoV-2). The copper and copper-nickel surfaces inactivated 99.9% of Ebola and Marburg viruses after 30 min, and the copper surfaces inactivated 99% of SARS-CoV-2 in 2 h. These data reveal that Ebola virus, Marburg virus, and SARS-CoV-2 are inactivated by exposure to copper ions, validating Luminore CopperTouch as an efficacious tool for infection control.

## INTRODUCTION

Emerging viruses continue to pose a major threat to public health worldwide, as demonstrated by two ongoing outbreaks. Viruses from the *Filoviridae* family have caused several outbreaks and epidemics in the past. Ebola virus caused an outbreak of Ebola virus disease in the West African nations Liberia, Sierra Leone, Guinea, Nigeria, Senegal, and Mali in 2014 to 2015, with a current outbreak in the Democratic Republic of the Congo (1, 2). More recently, severe acute respiratory syndrome coronavirus 2 (SARS-CoV-2) emerged from a seafood market in Wuhan, China, and has since spread quickly around the world.

Many infectious microorganisms can survive for days or weeks after landing on a surface, presenting a tremendous challenge for infection control. In one study, Zaire Ebola virus and Lake Victoria Marburg virus were shown to survive in liquid medium at titers above detectable limits for up to 46 days, although the number of viable particles decreased over that time (3). When dried on solid surfaces, such as rubber, glass, or plastic, Ebola virus survives at detectable levels for 5.9 (4) to 14  (3) days. SARS-CoV-2 can survive on plastic or stainless steel surfaces for up to 72 h and on copper for 4 h (5).

Because of the threat of fomite transmission of these viruses, attention has turned to the feasibility of self-sanitizing surfaces. Copper exhibits a self-sanitizing characteristic, but its weight and bulk have limited its use. To circumvent these challenges, antimicrobial copper coatings have been developed. Luminore CopperTouch is an EPA (Environmental Protection Agency)-registered antimicrobial copper surface. Copper and copper-nickel formulations are currently registered and available for application in hospitals, doctor’s offices, airports, buses, trains, and other heavily trafficked areas. These antimicrobial coatings greatly expand the possibility of using copper within the transportation and health care settings, particularly for high-touch surfaces, to reduce the spread of infection.

Several studies have demonstrated the effect of copper in reducing the bacterial burden in hospitals and health care facilities. Salgado et al. (6) reported a significant reduction in hospital-acquired infections and/or methicillin-resistant Staphylococcus aureus or vancomycin-resistant *Enterococcus* colonization for patients who received treatment in intensive care unit (ICU) rooms with copper alloy surfaces compared with those treated in standard ICU rooms. Viruses, including influenza A virus (7) and norovirus (8, 9), both of which are inactivated by copper, are susceptible to copper surfaces as well. Solid copper surfaces have also been shown to inactivate SARS-CoV-2 (5).

### Exposure of filoviruses to copper and copper-nickel sprayed surfaces.

Luminore is a polymetal alloy that is 85% copper, and the copper-nickel alloy is a proprietary blend containing 62.5% copper. For surfaces, 1-mm-thick 1- by 1-cm coupons were sprayed with the substance. All surfaces were disinfected with CaviCide followed by 70% ethanol. Stainless steel type 304 was used as the experimental control because it is biologically inert and has no known antimicrobial effect. The coupons of nonmetal surfaces were polyvinyl chloride (PVC). The sham metal surface was spray-painted stainless steel (Krylon fine paint).

All work with filoviruses was conducted by approved personnel under approved protocols at the Galveston National Laboratory, a biosafety level four (BSL-4) laboratory registered with the Centers for Disease Control and Prevention Select Agent Program, the University of Texas Medical Branch in Galveston, TX, in compliance with all regulations therein. Zaire Ebola virus (EBOV; stock) and Angola Marburg virus (MARV; stock) were utilized. The viral load consisted of 10^5^ plaque-forming units (PFU) and viral suspensions maintained in growth medium solution (Dulbecco’s modified Eagle’s medium with 1× l-glutamine, 1× pen/strep, 1% MEM vitamins, and 10% fetal bovine serum). Vero E6 cells were grown to 70% to 80% confluence in 6- or 12-well plates. Ten 1-μl drops (MARV) or one 10-μl drop (MARV and EBOV) of viral suspension was dispensed on copper, copper-nickel, or sham coupons (as described above) or on uncoated metallic or plastic surfaces as controls, with a subsequent 30-min incubation at room temperature. The drops were then collected with 100 μl of fresh dilution medium. Medium was pipetted on the dried virus spot and spread around the coupon to ensure maximum collection of deposited virus. This was then titrated on Vero E6 cells using a standard plaque assay technique (0.5% agarose overlay). Inoculated Vero E6 cells were incubated at 37 ± 2°C with 5% CO_2_ for 7 (MARV) or 10 (EBOV) days. The titrations were subsequently fixed with formalin. To enumerate the plaques, the wells were stained with neutral red or crystal violet solutions using standard procedures. To minimize the extent of work performed in the BSL-4 laboratory and the production of waste, experiments were conducted in duplicate.

**FIG 1:**
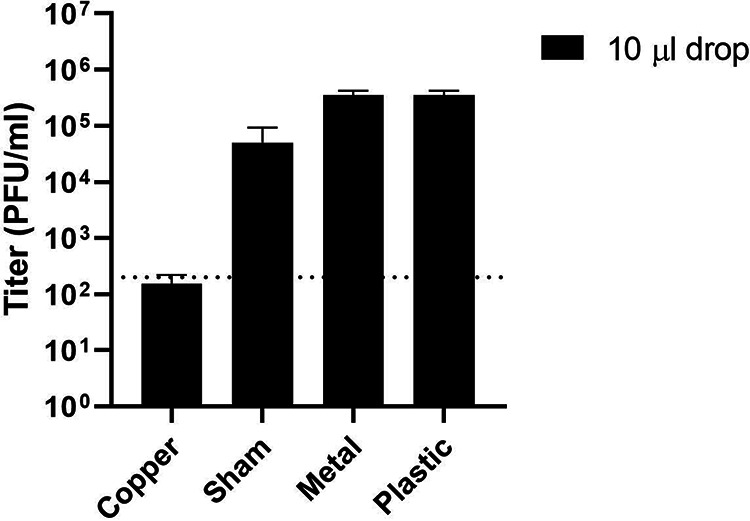
Ebola virus (Zaire) and Marburg virus (Angola) are inactivated on copper and copper-nickel surfaces. EBOV (10-μl drop) (top) and MARV (10-μl drop or 10 × 1-μl drops) (bottom) viruses were exposed to the indicated surfaces for 30 min and were then used to infect Vero E6 cells for titration. The average from two experiments is shown, with error bars representing the range. Dotted lines, limit of detection.

**FIG 2:**
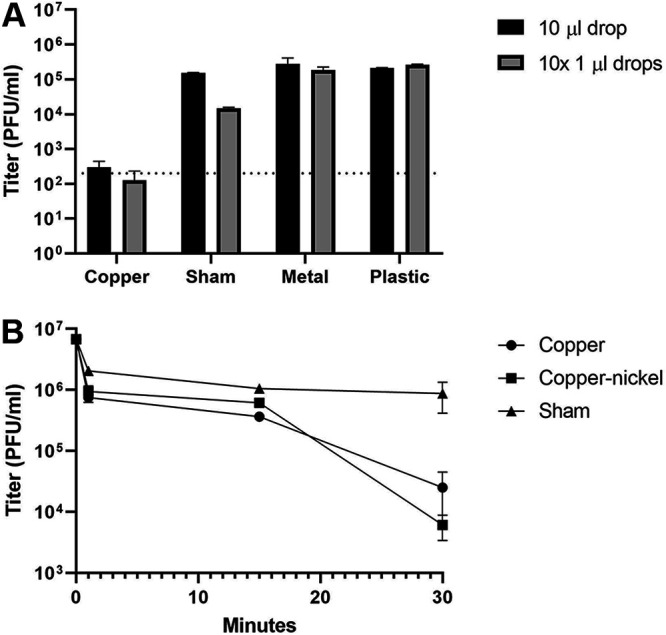
Ebola virus is inactivated within 30 min of exposure to copper and copper-nickel surfaces. Suspensions of Zaire Ebola virus (EBOV) were exposed to each surface for the indicated amount of time, and the exposed viruses were used to infect Vero E6 cells for titration. Copper, copper-nickel, and sham surfaces were compared for up to 30 min. The average from two experiments is shown, with error bars representing the range.

We observed a 99.9% reduction in viral titer on copper surfaces for MARV and EBOV relative to the sham surfaces after 30 min, approaching the limits of assay detection (Fig. 1). To determine how rapidly copper surfaces can inactivate viral particles, we measured EBOV titers using plaque assays on Vero E6 cells after viral suspensions had been exposed to surfaces for 1, 15, or 30 min at room temperature (Fig. 2). At 1 and 15 min, the viral titers decreased by ∼0.5 log-fold relative to the sham surface. After 30 min, the copper surface had reduced the viral load by ∼1.5 log-fold, corresponding to a 97% reduction in viral titers. The copper-nickel surface displayed a decrease of 2.3 log-fold, corresponding to a reduction of >99% in viral titers. These data suggest that viral inactivation occurs within 15 to 30 min and that the viral load is reduced by nearly 99% within that time.

### Exposure of SARS-CoV-2 to copper-sprayed surfaces.

**FIG 3:**
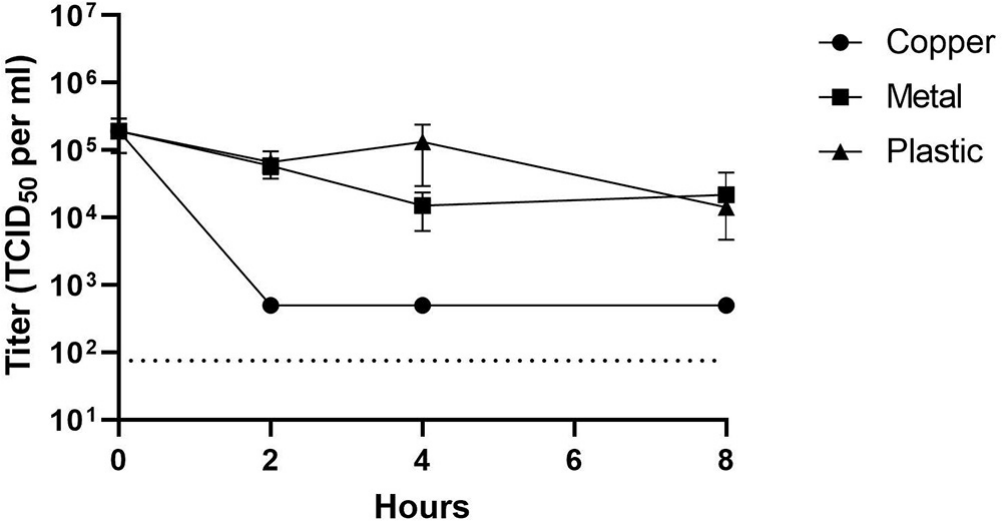
SARS-CoV-2 is inactivated within 2 h of exposure to copper-coated surfaces. One 10-μl drop of SARS-CoV-2 was added to the indicated surfaces for the denoted amount of time. The samples were collected with medium and were used to immediately infect Vero CCL-81 cells for calculating the viral titer via TCID_50_. Data shown represent the average titers for experiments performed in triplicate. The error bars display the standard distribution. Dotted line, limit of detection.

SARS-CoV-2 (USA-WA1/2020) was obtained from the World Reference Center for Emerging Viruses and Arboviruses (WRCEVA). All experiments with SARS-CoV-2 were approved and conducted by certified personnel in approved BSL-3 facilities at the University of Texas Medical Branch. Vero CCL-81 cells were grown to 85% to 95% confluence in 96-well plates. One 10-μl drop of SARS-CoV-2 stock (5 × 10^5^ 50% tissue culture infective dose [TCID_50_]/ml) was added to copper-coated or uncoated metallic or plastic surfaces as controls and incubated for the indicated duration at room temperature. These experiments were conducted in triplicate. The drops were collected with 90 μl of fresh dilution medium after 2, 4, or 8 h and titrated using a TCID_50_ assay. Regardless of the incubation time, the copper-coated surfaces reduced the viral titers by >2-fold, approaching the limit of detection (Fig. 3). This value corresponds to a reduction of >99% in viral titers. Exposure to copper surfaces for 2, 5, or 30 min did not significantly lower viral titers (data not shown).

The mechanism by which copper inactivates microorganisms is not completely understood. Pastor et al. (10) demonstrated that copper can donate and accept single electrons, which produces reactive oxygen species and free radicals, causing cell death. The ability of copper to inhibit bacteria involves the rapid degradation of genomic and plasmid DNA. One study further showed that DNA degrades rapidly on copper surfaces (11). Thus, it is plausible to hypothesize that the genomes of these viruses are disrupted by the free radicals produced on contact with copper ions.

Research on the antiviral and antibacterial properties of copper alloys and other metals (e.g., silver, iron) has increased tremendously over the past 5 years. Sehmi et al. (12) developed a method for encapsulating silicone and polyurethane with copper, and both exhibited antibacterial activity. By comparing thick (100-μm) and thin (25-μm) rolled copper plates, researchers have noted that thin-rolled sheets are rougher in texture than thick-rolled sheets. However, the data comparing their relative effectiveness are contradictory. Whereas Zeiger et al. (13) demonstrated that rough deposited copper surfaces have more antibacterial activity than smooth surfaces, Yousuf et al. (14) showed that thin-rolled sheets have more potent activity and attributed this effect to the increased surface area of the rough surfaces. More recently, a hybrid coating containing silver, copper, and zinc cations was found to significantly reduce viral titers for HIV-1, human herpesvirus 1, dengue virus type 2, and influenza H1N1 virus (15). One of the likely reasons for the early inactivation of EBOV compared to SARS-CoV-2 has to do with the different structures of the viruses. EBOV is long and filamentous, whereas SARS-CoV-2 particles are spherical with spike proteins; therefore, EBOV has more surface area in contact with the copper surface, leading to early inactivation. The coronavirus spike proteins also increase the distance of the viral capsid (the active site for copper) from the contact surface of the copper surface, increasing the inactivation time. These new data may further support the use of copper-coated surfaces in hospitals and other public places as an additional infection control measure during ongoing and future epidemics.
